# *Smart Government Applications and their Role in Process Redesign*  Applied study at Al Ittihad Food Industries Company

**DOI:** 10.12688/f1000research.175451.1

**Published:** 2026-05-14

**Authors:** Basma Mahdi Hamad, Fatimah Hussein Kazim, Zainab Abd Ulrazzaq Abood

**Affiliations:** 1 Business Administration, University of Babylon, Babylon Governorate, Babylon Governorate, Iraq

**Keywords:** Production Management, Smart government practices, Redesigning production processes, Digital integration, Artificial intelligence, Smart governance.

## Abstract

Smart government is a relatively new concept that has attracted the attention of researchers in various fields, including information technology, public administration, and political science. However, its impact on industry and recent developments, particularly those stemming from the Fourth Industrial Revolution, remains unclear. This study aims to analyze the effect of smart government practices on redesigning production processes using a descriptive-analytical approach. A practical application was conducted at the oil extraction plant of Al Ittihad Food Industries Company. A questionnaire served as the primary data collection tool, supported by quantitative analysis using statistical methods. The questionnaire was administered to a sample of 150 managers, engineers, and production unit employees. The results demonstrated a significant impact of smart government practices on redesigning production processes, contributing tangibly to improved process performance, enhanced quality, and strengthened organizational factors that support redesign. This research provides a practical framework that industrial organizations can utilize to formulate more integrated strategies for achieving sustainable improvements in performance, quality, and organizational effectiveness.

## 1. Introduction

Over the last decade, manufacturing industries have been experiencing mounting pressures in their efforts to cope with technological and economic changes. Digital transformation and innovation are necessary conditions for a slight comparative advantage. A smart government is considered a core concept in new policies; it is a set of practices that integrate digital, artificial intelligence, and smart governance assumed to impact fundamentally the efficiency and flexibility of manufacturing sectors. It will also be considered as a strategic factor for organizations in the industrial sector that aims at improving performance, enhancing quality, and developing internal organizational factors to enhance sustainable competitiveness.

This research is based on an assumption that smart government eventually leads to redesigning production processes in the industrial sector through specific dimensions comprising performance, quality, and organizational factors. To support this hypothesis, a literature review was carried out concerning digital integration, artificial intelligence, and smart governance with process redesign. Interest in smart government practices found growing attention over the last few years due to trends toward digitization. However, most application-oriented studies apply such practices within government services, e-governance, and citizen interaction ( Jantanukul et al., 2025; Guenduez et al., 2018). The relationship between intelligent/smart government/process redesign in the industrial sector has not been given adequate attention. Most of the available literature discusses in detail the dimensions of digital technologies, artificial intelligence, and IoT on operational performance and production efficiency ( Ghobakhloo & Ching, 2019). Therefore, this is an integrated analysis yet to be conducted regarding the aspects of smart government—represented by digital integration, artificial intelligence, and smart governance—that contribute to holistic redesigning of production processes with performance, quality, and organizational factors. In addition to being scanty in the Arab industrial context that rarely addresses its issues systematically linking it to production redesign, this research attempts at bridging a knowledge gap through applied analytical framework linking research variables thereby adding scientific value by expanding existing literature scope as well as practical value through providing decision makers’ recommendations towards enhancing efficiency & competitiveness within industrial organizations.

The importance of this research can be highlighted from two main aspects. First, it attempts to fill the research gap between theoretical literature and empirical evidence on the applications of smart government as an influential factor in the redesign of production processes within industrial establishments among countries attempting to catch up with the digital economy. Second, this paper analytically provides ways for policymakers and factory managers on how smart government strategies could be made parallel with Industry 4.0 objectives of Competitiveness and Sustainable Productivity.

This research used a descriptive-analytical method in the industrial sector of the Union Food Industries Company. A questionnaire and semi-structured interview were applied to obtain primary information. Data were analyzed by SPSS through several statistical methods. The research paper is formulated based on the following main sections, including a review of related literature in the second section. The third section presents Research Methodology and finally fourth section presents Conclusions and Recommendations.

## 2. Theoretical framework

### 2.1. The concept of smart government

Artificial intelligence, Internet of Things, cloud computing, and big data analytics comprise components of the larger Fourth Industrial Revolution or Industry 4.0 that have affected different facets of life. Governments are now able to transition from offering digitized services to smart and proactive services due to such technologies. It has made it possible for government organizations to enhance their abilities at all levels regarding collecting information and analyzing it comprehensively so as to better understand citizens’ needs and interact with them more effectively and efficiently. In this regard, smart government represents a leading feature emanating from Industry 4.0 because according to Alzoubi et al., (2025), “the use of advanced technologies together with processing as well as analysis involving big data within public administration accelerating the pace towards digital transformation within this domain.” The core of Smart Government is the citizen, with digital platforms and new solutions as innovation-oriented resource allocation to enhance popular participation, improving efficiency in service delivery and transparency among the priorities ( Liang et al., 2023). Smart government also intends to foster an ecosystem for innovation and public participation based on informed, data-driven decision-making ( Al Rashdi, 2024). The concept of smart government is different from that of e-government. The main focus of e-government is the digitization of existing services and enhancing efficiency through the use of information and communications technology. Integrating services or using advanced technologies is not a requirement for this. In smart government, processes are simplified, thus making service delivery more efficient; hence, there is minimal citizen participation ( Sadykova & Galy, 2024). The concept of digital government transformation aims to comprehensively improve the quality of public services and responsiveness to them, focusing on comprehensive changes in government structures and practices, i.e., integrating digital tools and platforms for innovation across all government functions ( Alexakis, 2023).

Smart government highlights policies that greatly impact the business environment, especially the industrial climate by promoting innovation and providing competitiveness as well as driving economic growth in the country. It is realized through supporting facilitated by adopting high technologies leading to productivity and general efficiency improvement. Concerning high technology adoption, governments of such countries as China have placed their strategic investments in Internet of Things Technology, Artificial Intelligence, and Robotics Technologies which are considered highly important elements for modern manufacturing practices. For example, plans like Made in China 2025 are practically amalgamating a few very latest technologies within traditional industries towards their maximum productivity as well as enhancing overall corporate performance ( Tsymbal, 2023). This was also undertaken by the South African Government in encouraging competence among automobile producers using smart manufacturing technologies that bring about more efficiency at a lower cost unveiling an immediate necessity for government assistance in executing such systems of smart manufacturing ( Inyang et al., 2023). Adept policies facilitate the local content and attract investors while establishing a good environment. Other than that, they are ingredients for market performance and planar development of clusters that bring about innovation and productivity ( Ejaz, 2023) thus realizing economic steadiness via encouraging local manufacturing instead of importing. This shows another balance dimension which is the adoption of both policy support and private sector participation toward sustainable industrial development. The following paragraphs discuss the dimensions of smart government which include digital integration, artificial intelligence, and smart governance.

**Digital integration** Digital integration is the heart of a netted smart government that can provide more services through the use of network infrastructure and interoperability. Digital systems make it possible to easily share information among different departments, hence making applications on digital platforms simplifying public service-that is the smart government. Artificial intelligence spread, digital integration, and increasing interaction between citizens and governance work to enhance productivity, develop easy administrative procedures, and establish an accountable and accessible governance structure ( Bodemer, 2024; Vatamano & Tofan, 2025). Digital integration can be defined as the unification of digital systems, information, and technologies ensuring quick and truthful conveyance of details throughout the functional areas ( Awad, et al., 2020). The approach assists in connecting the holes among running & management methods however it’s also a base lead to reconstructing generation techniques raising usefulness versatility plus fast reply towards modifications According to ( Marcos et al., 2021), process redesign in industrial organizations cannot achieve tangible results in performance and quality unless there is an integrated digital infrastructure that supports communication between the various production stages. In the words of ( Abdel-Aty & Negri, 2024), the concept of a thin thread sheds light on the core of digital integration as it connects all stages of product manufacturing from design to production to maintenance. This connection allows for the flow of data that supports evidence-based decisions and opportunities to continuously improve quality while reducing production time. Research by Zhang & Zhang (2025) found that creating a wholly integrated digital government directly contributes to enhancing the smart transformation of industrial organizations by a digital environment supportive of the integration of systems and processes. Their results also indicated that digital integration not only improves production performance but enhances institutional innovation and the ability to develop sustainable operating models.

**Artificial intelligence** can increase operational efficiency by automating tax processes, reducing bureaucratic delays, and increasing accuracy ( Bodemer, 2024). Sensitive government data is critical and AI is being merged with cybersecurity technology to enhance threat detection and response capabilities ( Jha & Jha, 2024). Good governance plays a significant role in developing government policies on the safe use of AI to address risks related to data privacy and algorithmic bias ( Al Dajeh, 2024; Jha & Jha, 2024). Recent research highlights meaningful participation as an indispensable pillar in advanced e-government through the integration of smart governance concepts with AI. This provides opportunities for the government to improve its practices while pointing out issues concerning ethical considerations-in-legislation-and-in-need-for-strong-regulatory-frameworks-to-be-addressed-and-successfully-implemented.

**Smart governance** is a core component of smart government hence it underlies a set of digital policies and mechanisms that ensure transparency, accountability, and interactive engagement between organizations and stakeholders. Sun et al. (2023) Environmental governance-a form of regulatory governance-is a moderating factor in the relationship between digital transformation and high-quality development in industry, thus indicating the role that regulatory/governmental policies play in attaining high performance/quality ( Cohen, 1977). Zhi-mei et al. (2024) also pointed out elements crucial for effective smart governance: data quality and flow mechanisms with regulatory safeguards including legislation and regulatory support ( Marcos et al., 2021). Mora et al., (2023) too highlighted smart governance as a framework applicable to managing innovation within smart cities through developing policies/legislation/procedures supporting stakeholder engagement besides regulating roles/responsibilities/risk management. These aspects can be transferred within the context of production processes. In the same context, Singha et al. (2023) asserts that smart governance contributes to enhancing industrial innovation through participatory mechanisms that enable industrial organizations to integrate stakeholders into development and production processes, which supports the process of redesigning production processes in line with dynamic changes in the markets. The results of his study showed that organizations that adopted smart governance were more capable of conducting continuous improvement processes.

Finally, operational efficiency and government support and incentives in the form of grants or subsidies can be articulated in reducing the financial burden of adopting smart manufacturing. The transition will also be smooth if workers, who are eventually participants in the transformation toward a smart factory, acquiesce to upskilling programs that involve high-level training in intelligent technologies. This government practice is considered a good government practice because it encourages factories to reconstruct their production processes by supporting an enabling environment for transformation through the adoption of Industry 4.0 technologies ( Matošková et al., 2023). The government can set the necessary regulations on intelligent manufacturing, making sure that safety and quality standards are observed while encouraging factories to adopt sustainability practices as part of broader environmental policies. The government makes factories more flexible to market dynamics by supporting technologies which allow real-time monitoring and control, among other aspects ( Nugroho, 2024; Awad et al., 2024). Due to perceived complexity and costs in the implementation of smart technologies, some factories may be reluctant to re-design their operations. Such reluctance can hamper the overall progress towards smart manufacturing. Therefore, comprehensive support and clear communication with stakeholders or governments are still largely emphasized.

## 3. Process redesign concept

Business process redesign is defined as “a systematic approach for radical rethinking and comprehensive redesign of the organization’s core processes.” In practical terms, results are significant improvements in performance-in-cost reduction, quality improvement, speed of delivery, and customer satisfaction ( Rybchuk et al., 2023). Therefore, an analysis regarding present process performance, its weaknesses or failures in the process, and reimagining and developing from scratch the way business is conducted becomes pertinent. The changes can be extremely radical but focused on strategic goals and added value for customers. This represents a huge investment that must be accompanied by changes at the system level-roles, technology, and organizational culture. It is an approach to replace traditional ineffective or inappropriate processes concerning current or future challenges of the organization with new ones ( Ondov et al., 2022). Molina (2021) also highlights that this investment does not need to be large since short long benefits may be greater than the investment. He insists that an understanding and analysis of all main production activities must be included to achieve a redesign of the business. Therefore, current processes have to be analyzed and changed in order to improve efficiency and effectiveness ( Asadinia et al., 2024). Digital technologies also enable process redesign by providing organizations with digital capabilities they can use to enhance processes such as analytics and communication ( Kubrak & Milani, 2023).

The organizations involving enhancement of digital transformation mostly associate this with differentiating process improvement from redesigning the process. Process improvements can be defined as betterments added to the existing process by incremental changes, such as streamlining, removing unnecessary wastages, and inculcating quality ( Alshurideh et al., 2022). High-volume new technologies applied workflows without changing any structure for incremental gains in cost take redundancy out of the system. Process redesigns are addressed to obtain breakthrough results by fundamentally rethinking and restructuring the process—completely overhauling the process to accommodate new technologies and methods ( Kubrak & Milani, 2023). This kind of change ensures dramatic improvements in operational efficiency and quality ( Aljazzi et al., 2022). This clearly shows that process improvement can result in instant change while process redesign is necessary for organizations facing disruptive change because it permits a more profound adjustment to market requirements, innovation, and digital transformations. Process redesign is based on several dimensions, i.e. performance, quality, and organizational factors. The following sections elaborate on these dimensions.

One of the basic dimensions in process redesign happens to be the **performance** dimension with results and impacts of redesigned processes within organizational and operational levels assessed. Leggat et al. (2016) sayed that the performance dimension reveals output analyses, which show to what extent objectives have been attained, thus enabling unexpected post-implementation obstacles or challenges to be detected for sustainability through continuous improvement of change as well as better future development efforts oriented toward an organization and its stakeholders’ needs. Their study proved that changing workflows and procedures made work efficient and accelerated turnaround times, hence output increased. Automation of the value chain improved efficiency and minimized stock outs. This therefore shows that process redesign can be a powerful driver of improved operational performance in manufacturing organizations. According to Gross et al. (2020), understanding the performance dimension assists in better exploration of process design alternatives since they are evaluated based on the extent to which performance improvement has been achieved, thus enhancing decisions based on realistic data as well as accurate measurements. Tsakalidis & Vergidis (2021) noted that choosing appropriate performance metrics is an integral part of both strategy formulation for redesigning as well as evaluation thereof; it directly influences success or failure thereof.

**Quality** is a basic aspect of process redesign. It pertains to emphasizing improved standards of output quality and minimized defects during redesign. Other research reveals that an accredited quality management system, for example, ISO 9001, fosters deeper implementation of redesign by organizations which have undergone certification to implement process redesigns more actively compared with their counterparts without such certifications ( Raji’c et al., 2024). In industrial organizations’ context, the quality dimension seeks an alignment between the processes and TQM/Quality 4.0 practices aimed at enhancing product reliability as well as process safety. Hence it becomes a tool for integration of quality standards into redesign making sure new processes are more customer-oriented besides being defect free and better aligned to certification requirements.

The association of quality with production and the redesign of processes directly deliver on performance results. Quality increases waste and time of production, which automatically upgrade efficiency and productivity leading to customer satisfaction. Several researchers have spotted substantial effects that process redesign bears on productivity and efficiency through different case studies. A manufacturing case study ( Bhaskar, 2025) applied the “B2Lean” model, where BPR and Lean tools were used in integration pointed out by the researcher as a new methodology significantly enhancing operational efficiency with a high impact on reducing production cycle times besides increasing product quality; hence faster resource deliveries better allocations when processes are redesigned qualitatively minded. The quality dimension links improved customer satisfaction because high operational standards reduce complaints while increasing repeat customers. A study (Gomaa et al., 2024) applied the DMAIC methodology from Lean Six Sigma with process reengineering in an electrical control panel factory. Defect rates significantly dropped, by 17.1% to only 3.1% and annual customer complaints reduced by 45 to just 3. All of this demonstrates that improving quality directly contributes to increasing productivity and operational efficiency and reducing waste, thereby leading to better results for redesigning production processes.

One of the key pillars of industrial process redesign and its success is **organizational factors**. This refers to all aspects of an organization’s internal structure that could hinder the redesign of production processes, such as leadership and strategy, structure and governance, culture and change, human capabilities, and implementation and monitoring mechanisms. Recent literature shows that the success of process redesign is linked to the interconnectedness of these factors, not to a single factor ( Fetais et al., 2022a). An empirical study ( Vishvakarma et al., 2021) involving 131 organizations, categorized into strategic groups (conservatives and innovators), demonstrated that organizational strategy clearly influences the scope, profitability, and efficiency of process redesign, along with the direction of information flow (top-down or top-down), as well as organizational resistance to change during the redesign process implementation. Another study ( Fetais et al., 2022b) highlights the importance of organizational culture, leadership, and knowledge sharing in promoting process redesign. This study analyzed the relationship between process redesign and corporate culture in the manufacturing sector. Their study found that strategy, leadership, and knowledge transfer are closely related to the core process redesign factors, while some cultural attributes, such as team orientation and attention to detail, exhibit a moderate correlation. The least related was aggressiveness. Factors such as senior management commitment, organizational readiness for change, information technology capabilities, and human resource management have a significant positive impact on organizational performance, and organizational structure has a stronger impact on improving performance when a forward-thinking strategy is present within the organization ( Hameed et al., 2022).

To clarify the causal relationship between the study variables, and based on the theoretical arguments and discussions, and the applied evidence presented in previous studies, which show the importance of smart government practices as one of the main drivers of organizational change and operational transformation, and their contribution to enhancing operational efficiency, and promoting the ecosystems for innovation and public participation through data-driven decision-making ( Al Rashdi, 2024), which represents a set of digital initiatives, technologies and policies that aim to improve efficiency, transparency and speed of response within organizations ( Sun & Fang, 2023). It is evident that adopting these practices contributes to redesigning production processes and enhancing innovation, flexibility, and sustainability ( Ejaz, 2023). These practices can be viewed as the independent variable that directly impacts the management and operation of production activities. On the other hand, redesigning production processes represents a dependent variable, encompassing a set of procedures undertaken by organizations to re-engineer their operations ( Alshurideh et al., 2022). These procedures aim to increase operational efficiency, reduce costs, and improve quality and sustainability ( Asadinia et al., 2024). The causal relationship between the two variables can be explained by demonstrating that adopting smart government practices, with their various dimensions, can stimulate the redesign of production processes by simplifying procedures and enabling data-driven decision-making. This is an explanatory and influential factor in the extent to which organizations can redesign their processes to align with the requirements of new production conditions, including smart production, ultimately achieving the goals of digital transformation. This is the objective we aim to test and analyze in the company under study. A conceptual model for the research was developed, as shown in Figure 1.

**Figure 1.**
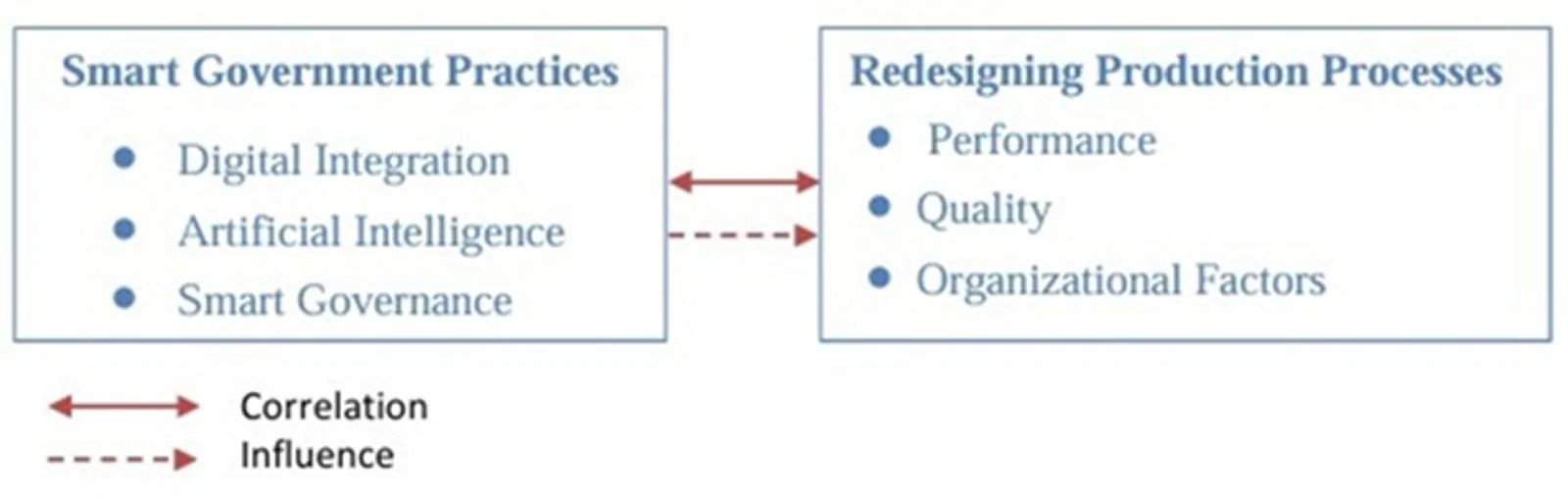
Conceptual model.

## 4. Research methodology

The descriptive-analytical approach was adopted, as it is characterized by its ability to describe the phenomenon as it exists in the field, and then analyze it to discover its patterns and the relationship between its variables. The aim of using this approach is to explore the relationship between the dimensions of smart government (digital integration, artificial intelligence, smart governance) and the redesign of production processes in its dimensions (performance, quality, organizational factors). This approach allows for the collection of quantitative and qualitative data about the nature of the target society and its scientific analysis in order to arrive at accurate results that contribute to explaining the phenomenon. A mixed methodology was adopted, where qualitative data were collected through questionnaires administered to managers and engineers working in production units. This brought the sample size to 150, which is purposive sampling. The purposive sampling technique was chosen because the study needs responses from those individuals who have adequate experience and knowledge about digital developments and variables and redesigning production processes so that more accurate data can be obtained.

The reason the descriptive-analytical approach was picked is that it best suits an investigation of phenomena intersecting technical and organizational dimensions. It provides great value in systematically analyzing complex phenomena tied to specific contexts, thereby enhancing credibility. Framing the study, collecting and interpreting data, and making an explicit contribution to knowledge are also enabled through this approach when applied to a practice-based research context ( Kearney, 2022). This is also true for the relationship between smart government and production process redesign. The selection is thus consistent with recent literature in the Industry 4.0 field advocating interpretive analytical approaches toward assessing effects brought about by smart policies and digital transformations regarding industrial efficiency ( Zhang & Zhang, 2025; Sharma & Chen, 2023).

Data were analyzed using the SPSS program, one of the most common tools in administrative and social research. This is due to its advanced capabilities for processing quantitative data accurately and objectively. A set of statistical methods appropriate to the nature of the study was used. This methodology contributes to providing a comprehensive and coherent analysis of the hypothesized relationships, while ensuring the accuracy of the results and their applicability to the manufacturing sector.

The research aimed to achieve the following objectives:

1. To identify the extent to which the company adopts smart government practices, such as digital integration, artificial intelligence, and smart governance, from the perspective of the research sample.
2. To explore the relationship between smart government practices and the redesign of production processes, based on the responses of the research sample.
3. To provide practical recommendations, based on the statistical analysis of the research sample’s responses, to enhance the effectiveness of smart government practices in improving the production process.

## 5. Empirical analysis and results

### 5.1. Reliability and validity of the research measuring tool

Reliability means consistency and stability of a scale. Therefore, if the same scale is used on the same sample repeatedly, it gives the same results. Hence, reliability means consistency and stability of the scale ( Sekrana, 2003: 203). One of the most widely used methods in testing the reliability of questionnaire items is Cronbach’s alpha coefficient. As noted by Sekrana (2003: 311), if the coefficient value is less than 0.60, then it shows low reliability of the employed scale while a value more than 0.70 can be taken as acceptable and above 0.80 as good.

Validity is about whether the scale measures what it purports to measure. In other words, it raises this question: Does the scale measure the phenomenon under study and not something else unrelated? ( Sekrana, 2003: 206). There are different types of validity used by researchers. One is content validity. Content validity depends on the explicit definition by the researcher of the variables regarding the research topic, which in turn is very much influenced by the quantity of information being examined ( Cooper & Schindler, 2014: 257). Table 1 presents reliability coefficients for each variable and its dimensions.

**Table 1.** Values of the reliability and validity coefficients for the dimensions of the research variables.

No.	Dimensions	Cronbach’s Alpha
**Smart Government Practices**		**0.789**
1	Digital Integration	**0.736**
2	Artificial Intelligence	**0.756**
3	Smart Governance	**0.761**
**Production Process Redesign**		**0.783**
1	Performance	**0.769**
2	Quality	**0.753**
3	Organizational Factors	**0.746**

Table 1 clearly indicates that the reliability and validity coefficients for the research variables smart government practices and the redesign of production processes, along with their respective dimensions—fall within statistically acceptable ranges. This suggests that the measurement scale employed for the research sections possesses a high level of reliability, thereby allowing researchers to confidently base their decisions on the forthcoming results.

### 5.2. Testing the normal distribution of data

Following the researchers’ validation of the data collection instrument through a stability test, it is important to note that the hypothesis testing in the present study relies on parametric statistics. This approach is grounded in the fundamental assumption that the data under analysis must exhibit a normal distribution. Should parametric methods be applied to data lacking this normality, the results derived from such tests would be deemed unreliable ( Field, 2009: 132). While statisticians have pointed out that concerns regarding the normal distribution of data may be alleviated when researchers utilize a sample size significantly larger than that of the research population ( Field, 2009: 329), to ensure the precision of the research findings, the data collected via the questionnaire underwent one of the key tests for normal distribution: the Kolmogorov-Smirnov test. This test indicates that if the sample size exceeds 35 individuals, the test value can be calculated using the following formula ( Cooper & Schindler, 2014: 623).

$$D=\frac{1.22}{\sqrt{n}}$$

Where n is the sample size here, and since the research sample size is (150) individuals, then the standard (D) value shall be (0.09). If the value of the Kolmogorov-Smirnov statistic is greater than or close to the standard (D) value at a significance level of 1%, this will mean that the data are normally distributed at this level. Hence, parametric statistical analysis tools can be applied and results assured. If data do not follow a normal distribution, then researchers fall back on non-parametric analytical tools. Table 2 below shows results for normal distribution tests by research variables and their dimensions.

**Table 2.** Testing the normal distribution of research variables data and their dimensions.

No.	Dimensions	Kolmogorov-Smirnov	Standard D value	Decision
**Smart Government Practices**		**0 *.*20**	**0.09**	Naturally distributed
1	Digital Integration	0.17		
2	Artificial Intelligence	0.13		
3	Smart Governance	0.14		
**Production Process Redesign**		**0.19**		
1	Performance	0.15		
2	Quality	0.12		
3	Organizational Factors	0.10		

It is evident in Table 2, the data of research variables at both sub-level and overall levels i.e. smart government practices and redesigning production processes fall under normal distribution, hence eligible to use parametric tools for analysis.

Diagnosis and description of the research variables: This paragraph seeks to state, analyze, and construe the results of responses by members of the research sample regarding the paragraphs in the questionnaire by viewing values of weighted arithmetic means, relative importance, standard deviations, and coefficients of variation for each paragraph relating to variables within this study.

The study defined the level of responses by mean averages, i.e., to what category they belong, and since the study model adopts a five-point scale (strongly agree - strongly disagree), there are five categories wherein the mean averages fall. The length of the range is determined to ascertain the category (5–1 = 4). The length of the range is then divided by the number of categories (5) (4 ÷ 5 = 0.80). Thereafter, (0.80) is added to the lower limit of the scale (1) or subtracted from its upper limit (5).

**Table:** 

1–1.80	Very Low
1.81–2.60	Low
2.61–3.40	Moderate
3.41–4.20	High
4.21–5.00	Very High

**Source:** Dewberry, Chris, (2004). Statistical Methods for Organizational Research:Theory and Practice. Published by Taylor & Franci.

This aspect will be addressed in the following paragraphs:

Presentation, analysis, and interpretation of the responses of the research sample individuals regarding smart government practices: The paragraphs of this variable will be addressed by extracting the values of the weighted arithmetic means, relative importance, standard deviations, and calculated coefficients of variation, whether at the partial or total level, as shown below.

Table 3 results indicate that the smart government practices attained an arithmetic mean of 3.25 with a standard deviation of 1.02 at a relative importance rating of 65%. This therefore means that smart government practices will simplify administrative procedures by reducing bureaucracy hence making it easy for business organizations to operate. It increases transparency and accountability to investors confidence as well as reduce corruption. Among other benefits, it offers accurate analytical tools supporting strategic decisions thereby bringing efficiency plus offering innovation to private sectors. The following is an explanation of the dimensions of smart government practices:

- **Digital Integration:** At the aggregate level, the dimension achieved a weighted mean of (3.07), meaning it falls within the “moderate” category. The relative importance reached (61%), while the standard deviation was (0.99). From the above, the importance of digital integration as a dimension of smart government practices is of great value to business organizations. This integration contributes to improving operational efficiency and reducing red tape by linking government digital systems with those of private institutions, facilitating rapid and transparent access to accurate data and information. Digital integration also enhances the ability to make informed decisions based on reliable analysis, creating a more flexible and responsive business environment to economic and technological changes.
- **Artificial Intelligence:** At the aggregate level, the dimension achieved a weighted mean of (3.10), meaning it falls within the “moderate” category, while the relative importance reached (62%), and the standard deviation was (0.94). From the above results, it is clear that artificial intelligence has a significant impact on enhancing the efficiency and effectiveness of business organizations. By analyzing big data quickly and accurately, artificial intelligence can enable organizations to make informed decisions, improve strategic planning processes, and provide integrated and higher-quality services to citizens and customers. It also contributes to enhancing transparency and oversight of government procedures, reducing bureaucracy and creating a more flexible and competitive business environment.
- **Smart Governance**: At the overall level, the dimension achieved a weighted arithmetic mean of (3.14), meaning that it falls within the (moderate) category, while the relative importance reached (63%), while the standard deviation value was (0.99), while the coefficient of variation percentage reached (31.52%). From the above, we conclude that smart governance contributes to enhancing trust between the public and private sectors, by ensuring integrity and accountability, and opens the way for innovation and the development of sustainable competitive strategies, which makes it a pivotal element in supporting the growth and efficiency of business organizations in light of the modern digital economy.

**Table 3.** Descriptive statistics for the dimensions of smart government practices n = 150.

No.	Dimensions	Weighted mean	Standard deviation	Relative importance%
1	Digital Integration	3.07	0.99	61
2	Artificial Intelligence	3.10	0.94	62
3	Smart Governance	3.14	0.99	63
**Smart Government Practices**		**3.25**	**1.02**	**65**

Presentation, analysis, and interpretation of the responses of the research sample individuals regarding the redesign of production processes: The paragraphs of this variable will be addressed by extracting the values of the weighted arithmetic means, relative importance, standard deviations and calculated variation coefficients, whether at the partial or total level, as shown below:

As seen in Table 4, this dimension scored an arithmetic mean of 3.06 with a standard deviation of 0.99 at 61% relative importance, redesigning production processes accelerates the organization’s ability to keep up with market changes and fulfill new needs of customers as well as support innovation through the integration of modern technologies with effective management methods. Thus, redesigning production processes is not an exercise that is limited to improving current performance only but rather should be viewed as a strategic tool for developing competitiveness and enhancing business sustainability in a dynamic and changing economic environment. Below is an explanation of the dimensions of redesigning production:

- **Performance:** At the aggregate level, this dimension got a weighted mean of 2.91 which falls within the moderate category, also relative importance reached 58% with a standard deviation value of 1.01. From the above, focusing on performance will enable organizations to be able to respond rapidly to market changes and perform innovation in their products and services. It supports strategic decisions at different levels by relying on accurate and realistic data about the progress of operations. Performance from this perspective is an essential tool for achieving integration between technology, human resources, and operational processes that institutionalizes survival plus development of an institution within competitive and changing business environments.
- **Quality:** At the aggregate level, the dimension achieved a weighted mean of (2.93), meaning it falls within the “moderate” category. The relative importance reached (59%), while the standard deviation was (0.99). The above results clearly show quality as the deciding factor in enhancing operational efficiency to achieve customer satisfaction. Waste and mistakes will be minimized by emphasizing quality, effectiveness of the resource used will be maximized hence having a positive impact on cost, time, and productivity. The organization can use this as a competitiveness enhancer whereby consistent products and services that meet customer expectations result in increased customer loyalty accompanied by an improved organization’s standing in the market.
- **Organizational factors**: At the aggregate level, this dimension achieved a weighted mean of (3.19), which falls within the “moderate” category while relative importance reached (64%) with a standard deviation of (1.04). This demonstrates its direct contribution to improving performance efficiency and achieving strategic objectives. It includes the structure of authority and responsibility distribution, patterns of coordination between various units, modes of decision-making, and an organizational culture fostering cooperation and innovation. Effectively organizing these factors enables the organization to reduce waste, accelerate the flow of operations, and improve the quality of products and services. It also enhances the ability to respond quickly to market changes and customer needs, making redesigning production processes more effective and sustainable from an operational and strategic perspective.

**Table 4.** Descriptive statistics for the dimensions of redesigning production processes n = 150.

No.	Dimensions	Weighted mean	Standard deviation	Relative importance%
1	Performance	2.91	1.01	58
2	Quality	2.93	0.99	59
3	Organizational Factors	3.19	1.04	64
**Production Process Redesign**		**3.25**	**3.06**	**0.99**

### 5.3. Testing the hypotheses of association and influence between the research variables

It aims to test the correlation and influence relationships between the research variables, as the correlation and influence relationships will be tested at the level of the sub-hypotheses that emerged from the main hypotheses, as well as testing the correlation and influence relationships at the overall level through the use of the simple correlation coefficient (Pearson) and the simple linear regression coefficient.

Testing the first main hypothesis related to the relationship between smart government practices and redesigning production processes (there is a significant correlation between smart government practices and redesigning production processes).

Three sub-hypotheses branched out from it, as shown below:

1. There is a significant correlation between digital integration and the redesign of production processes in all its dimensions.
2. There is a significant correlation between artificial intelligence and the redesign of production processes in all its dimensions.
3. There is a significant correlation between smart governance and the redesign of production processes in all its dimensions.

Table 5, shows the simple correlation coefficient matrix (Pearson) between these variables and their dimensions. Before testing this hypothesis, the table also indicates the sample size (150) and the type of test (2-tailed). The abbreviation (Sig.) in the table refers to the significance test of the correlation coefficient. If a (*) mark appears on the correlation coefficient, this means that the correlation is significant at the (5%) level, while if a (**) mark appears on the correlation coefficient, this means that the correlation is significant at the (1%) level. The strength of the correlation coefficient is judged in light of Cohen’s rule (1977: 79–81), as follows:

✓ Low correlation: If the correlation coefficient value ranges between 0.10 and 0.29.
✓ Moderate correlation: If the correlation coefficient value ranges between 0.30 and 0.49.
✓ Strong correlation: If the correlation coefficient value ranges between 0.5 and 1.

**Table 5.** Testing the first main hypothesis (correlation hypothesis).

Smart Government Practices	Digital Integration	Artificial Intelligence	Smart Governance		
0.52 **	0.32 **	0.51 **	0.50 **	**Pearson Correlation**	Production Process Redesign
0.000	0.000	0.000	0.000	**Sig. (2-tailed)**	
150	150	150	150	**n**	

** Correlation is significant at the 0.01 level (2-tailed).

Table 5 shows the correlation matrix that tested the first main hypothesis and its derivative hypotheses, that there are strong positive correlations (because its value is greater than 0.50) and significant at the level of (1%) between smart government practices and the variable of redesigning production processes, as the values of the simple correlation coefficients between these dimensions reached, as the correlation coefficient between smart government practices and redesigning production processes reached a value of (0.52) at the significance level of (1%), and it is considered a strong relationship in light of Cohen’s rule.

At the dimension level, the strongest correlation was between the artificial intelligence dimension and the redesign of production processes, as the correlation value between them reached (0.51) at a significance level of (1%), which is considered a strong direct relationship according to Cohen’s rule. The weakest correlation was between the digital integration dimension and the redesign of production processes, as the correlation value between them reached (0.32), which is considered a medium correlation according to Cohen’s rule.

The results indicate a positive correlation between smart government practices and the redesign of production processes in business organizations. Smart government enables the use of digital technology and big data to improve the efficiency of administrative procedures and facilitate access to necessary information. This enhances organizations’ ability to adopt innovative strategies to redesign their production processes, including streamlining processes, reducing waste, and accelerating the production cycle. Furthermore, smart policies contribute to providing a supportive legislative and regulatory environment, increasing organizations’ flexibility and ability to respond to market changes more quickly, thereby enhancing production performance and achieving a sustainable competitive advantage.

Based on the above, the first main hypothesis and its sub-hypotheses are accepted, which state that “there is a significant correlation between smart government practices and the redesign of production processes”.

Testing the second main hypothesis related to the influence relationship between smart government practices and the redesign of production processes (there is a significant influence relationship between smart government practices and the redesign of production processes).

Three sub-hypotheses branched out from it, as shown below:

1. There is a significant influence of digital integration on the redesign of production processes across all dimensions.
2. There is a significant influence of artificial intelligence on the redesign of production processes across all dimensions.
3. There is a significant influence of smart governance on the redesign of production processes across all dimensions.

It is clear from the results of Table 6 that the regression coefficient of the smart government practices variable on the redesign of production processes reached (0.52), which means that if the smart government practices change by one unit, the redesign of production processes will increase by (52%), noting that the effect is significant because the calculated (t) value of (10.28) is significant at the (0.000) level.

**Table 6.** Estimating the simple linear regression relationship between smart government practices and redesigning production processes.

Dependent variable/Independent variable	Production Process Redesign					
	β	R ^2^	T	Sig.	F	Sig.
Smart government practices	0.52	0.27	10.28	0.000	26.21	0.000

It is also noted that smart government practices explain (27%) of the changes in the redesign of production processes, while the remaining percentage is due to other variables outside the current research model. Note that the estimated model is generally significant because the calculated (f ) value of (26.21) is significant at the (0.000) level.

The results indicate that smart government practices promote the redesign of production processes in business organizations by improving information flow and supporting strategic decision-making. Digital technologies, such as e-services and big data analytics, provide a flexible and transparent regulatory environment that enables organizations to restructure their operations to achieve greater efficiency, reduce waste, and foster innovation, contributing to increased competitiveness and sustainability.

Accordingly, and based on the above, the second main hypothesis is accepted, which states: “There is a significant influence relationship between smart government practices and the redesign of production processes”.

Table 7 shows the following:

A. The regression coefficient was (0.32), meaning that if the dimension changed by one unit, the production process redesign variable would increase by (32%). Note that the effect was significant, as the calculated (t) value of (8.26) was significant at the (0.000) level.
B. The coefficient of determination (R2) was approximately (0.10), meaning that the dimension explains (10%) of the changes occurring in production process redesign, while the remaining percentage is due to factors other than those included in the current model.
C. We find that the calculated (F) value of (15.82) is significant at the (0.000) level. Therefore, we note that the estimated model is significant overall.

**Table 7.** Estimating the simple linear regression relationship between Digital integration and redesigning production processes.

Dependent variable/Independent variable	Production Process Redesign					
	β	R ^2^	T	Sig.	F	Sig.
Digital Integration	0.32	0.10	8 *.*26	0.000	15.82	0.000

Digital integration in smart government practices is a powerful factor in redesigning the production processes of business organizations. It enables efficient exchange of information and knowledge between different units, which contributes to streamlining procedures, improving performance, increasing organizational flexibility in responding to market changes, enhancing innovation, and achieving added value in production processes. Therefore, the researchers concludes from analyzing the results of Table 7 that the alternative hypothesis, which states, “There is a significant influence of digital integration on the redesign of production processes”, is accepted.

Table 8 shows the following:

A. The regression coefficient was (0.51), meaning that if the dimension changed by one unit, the production process redesign variable would increase by (51%). Note that the effect was significant, as the calculated (t) value of (11.22) was significant at the (0.000) level.
B. The coefficient of determination (R2) was approximately (0.27), meaning that the dimension explains (27%) of the changes occurring in production process redesign, while the remaining percentage is due to factors other than those included in the current model.
C. We find that the calculated (F) value of (21.13) is significant at the (0.000) level. Therefore, we note that the estimated model is significant overall.

**Table 8.** Estimating the simple linear regression relationship between artificial intelligence and redesigning production processes.

Dependent variable/Independent variable	Production Process Redesign					
	β	R ^2^	T	Sig.	F	Sig.
Artificial Intelligence	0.32	0.27	11.22	0.000	21.13	0.000

Artificial intelligence, as one of the dimensions of smart government practices, directly affects the redesign of production processes in business organizations, by enabling them to use accurate data and analysis to improve operational efficiency, reduce costs, and increase flexibility and quality, which enhances competitiveness and responsiveness to market variables. Therefore, the researcher concludes from the analysis of the results of Table 8 that the alternative hypothesis, which states “there is a significant influence relationship between artificial intelligence and the redesign of production processes”.

Table 9 shows the following:

A. The regression coefficient was (0.50), meaning that if the dimension changed by one unit, the process redesign variable would increase by (50%). Note that the effect was significant, as the calculated (t) value of (9.64) was significant at the (0.000) level.
B. The coefficient of determination (R2) was approximately (0.25), meaning that the dimension explains (25%) of the changes occurring in process redesign, while the remaining percentage is due to factors other than those included in the current model.
C. We find that the calculated (F) value of (19.73) is significant at the (0.000) level. Therefore, we note that the estimated model is significant overall.

**Table 9.** Estimating the simple linear regression relationship between smart governance and redesigning production processes.

Dependent variable/Independent variable	Production Process Redesign					
	β	R ^2^	T	Sig.	F	Sig.
Smart Governance	0.50	0.25	9.64	0.000	19.73	0.000

Smart government practices contribute to enhancing the effectiveness of redesigning production processes in business organizations by providing an integrated digital environment to support information exchange and data-driven decision-making, which increases operational flexibility, reduces costs, and enhances innovation and quality. This indicates a direct positive impact of smart governance on the development of production processes. Therefore, the researchers concludes from analyzing the results of Table 9 that the alternative hypothesis, which states, “There is a significant influence relationship between the smart governance dimension and the redesign of production processes”, is accepted.

## 6. Discussion

In this study, researchers experimentally examined the impact of smart governance practices, encompassing digital integration, artificial intelligence, and smart governance, on process redesign. This section of the study discusses the findings and their implications. The results demonstrated that smart governance practices, in all their dimensions, had a clear positive impact on the redesign of the production processes of the sample company. This impact is evident first in the technical dimension, related to infrastructure and digital platforms, and second in the organizational dimension. This effect contributes to improving production processes in terms of efficiency and structure.

It is clear that the study’s findings align with those of previous research, such as Ghobakhloo et al. (2019), which demonstrated that the application of Fourth Industrial Revolution technologies in the digitization of the industrial sector, such as Artificial Intelligence, Big Data Analytics, and the Industrial Internet of Things (IIoT), plays a significant role in enhancing and improving the efficiency of manufacturing processes. Similarly, the study by Sadykova and Galy (2024) confirmed that increased efficiency in public services provided by governments is due to their adopted digital transformation strategies, along with increased citizen satisfaction. This study also emphasized the importance of strategic leadership and the development of the necessary frameworks for successful digital transformation.

The current study has highlighted the positive impact of digital governance on operational performance, based on the achieved results. These results align with the study by Zhang and Zhang (2025), which found that the Chinese government’s digital transformation initiatives have fostered economic development and prosperity. It’s also worth noting the contextual and methodological differences in this study. For example, from an innovation perspective, some studies have focused on smart city governance, such as Mora et al. (2023). This context differs from our study, as Mora et al. discussed urban transformations, while our study adopted a field-based approach, selecting an industrial company and relying on questionnaire responses from the research sample to measure smart government practices and their direct impact on the company’s internal production processes. Furthermore, previous studies have addressed specialized topics in technologies related to artificial intelligence or government service delivery within regional contexts. This explains our study’s findings and some of the differences from previous studies, as well as our reliance on the local industrial context.

It is also important to clarify the differences in impact magnitude and statistical significance due to contextual and methodological variations. While there is general agreement that digitalization and smart government lead to improved production processes, the discrepancies stem from differing research environments, ranging from the level of development of government infrastructure to the policies adopted. For example, the study by Sadykova & Galy (2024) addressed digital government and its impact on service delivery at the national level, whereas our study focused specifically on the internal organizational impact of a selected company.

Finally, the results of our current study confirm the general trend established by previous research, by providing empirical evidence in a local industrial environment that has not been extensively studied before, regarding the research variables. The results showed that smart government, with its dimensions addressed in the study (digital integration, artificial intelligence, and smart governance), is not merely a theoretical concept, but plays a pivotal role in operational efficiency. Therefore, this study contributes to enriching the scientific understanding of the operational performance of industrial companies in developing environments and the extent to which it is affected by digital governance.

## 7. Conclusions and recommendations

Adopting smart government practices by their three dimensions: digital integration, artificial intelligence, and smart governance turned out to be the main supporting factors in redesigning production processes in the industrial sector. This study analytically proves that these dimensions are directly related to raising quality levels and improving operational performance as well as organizational factors regarding flexibility and sustainability. More so, digital integration is found by this research to be one of the major factors in process redesign since it can make the process flow more efficient hence reducing completion time and increasing resource efficiency. The findings further support the fact that artificial intelligence will always stand as a pillar of strength through support rendered in real-time monitoring of production processes, failure prediction, and exact quality standard controls. Smart governance, supported by transparency accountability elements and digital strategies, leads to be a great driving force for sustainable organizational change in industrial organizations because it opens up the gate to build an organizational culture through flexibility, innovation, and continuous improvement.

This study forms part of contemporary literature by empirically validating the claim that government policies do redesign processes. It further emphasized that transformations would only be successful not solely dependent on digital technology adoption but also an alteration of administrative and organizational systems that would sustain added value to industrial organizations. Hence, such industrial organizations that wish to improve their competitiveness amidst global changes and transformations should have within their set of policies an integrated approach wherein smart government policies are combined with production process redesign to fit into efficiency, quality, and internal organization requirements.

The results attained advocate for augmenting digital integration by investing in digital infrastructure and connecting the operational plus administrative systems of industrial organizations within an integrated framework that guarantees information flow at all levels of accuracy and effectiveness in decision-making. A plan to monitor production lines with artificial intelligence applications as well as a plan to predict early failure, equally plans regarding more quality management improvements will directly play a part in raising efficiency as well as in the reduction of waste. This is achievable through enabling digital transformation with a flexible governance system supported by digital data to ensure that the compliance process is industrial plus building trust among all stakeholders. Redesigning operations which suggest integration between performance quality and organizational factors directed toward long-term competitive advantage and sustainability.

## Ethical component

This study involved human participants and was conducted in accordance with accepted ethical research standards. Ethical approval was obtained from the Scientific Research Ethics Committee, University of Fallujah, Iraq (Approval No. HOF.HUM.2025.001). Written informed consent was obtained from all participants prior to their participation. All participants were informed about the purpose of the study, their voluntary participation, their right to withdraw at any time, and the confidentiality of their data.

## Informed consent

Written informed consent was obtained from all individual participants prior to their participation in the study. All participants were informed about the purpose of the research, their right to withdraw at any time, and the confidentiality of their data.

### Reporting guidelines

This study is an observational survey-based research and follows the STROBE reporting guidelines. No CONSORT or ARRIVE checklists are required as the study does not involve clinical trials or animal experiments.

## Data Availability

Repository name: Smart Government Applications and their Role in Process Redesign: Applied Study at Al Ittihad Food Industries Company. https://doi.org/10.5281/zenodo.18929706 ( Hamad, 2026). The project contains the following underlying data: • Dataset (raw questionnaire data used for all statistical analyses reported in the study). Figures and Tables Repository name: Smart Government Applications and their Role in Process Redesign: Applied Study at Al Ittihad Food Industries Company. https://doi.org/10.5281/zenodo.18929706 ( Hamad, 2026). The project contains the following extended data:
• Questionnaire (survey instrument used for data collection). Data are available under the terms of the Creative Commons CC0 1.0 Universal Public Domain Dedication.
