# Supplementary material for: Smart Government Applications and their Role in Process Redesign  Applied study at Al Ittihad Food Industries Company
Source: F1000Res. 2026 May 14;15:736. [Version 1] doi: 10.12688/f1000research.175451.1 (PMC13653099; doi:10.12688/f1000research.175451.1)
Supplement: Peer review history

# Reviewer report: Reviewer response for version 1

**DOI:** 10.5256/f1000research.193434.r525118

**Published:** 2026-10-09

**Authors:** M. A. K. Harahap

**Affiliations:** 1 Simalungun University, Pematangsiantar, Indonesia

**Dear Authors,**

 This manuscript examines the relationship between smart government practices—digital integration, artificial intelligence, and smart governance—and production process redesign in an industrial company. The topic is relevant and potentially valuable, particularly because it extends the discussion of smart government beyond conventional public-sector applications into an industrial setting. The availability of the underlying dataset and questionnaire is also a positive aspect that supports transparency and reproducibility.

 However, several methodological and statistical issues should be addressed before the manuscript can be considered fully scientifically robust.

1. **Conceptual framework:** Please clarify how “smart government practices” are operationalized at the firm level. The distinction between government-level digital practices and the company's internal digital transformation should be made explicit.
2. **Research design:** The manuscript describes the study as employing a mixed methodology and mentions semi-structured interviews, but the reported analysis is essentially questionnaire-based and quantitative. Please clarify the qualitative component and explain its collection, analysis, and integration, or revise the methodological description accordingly.
3. **Sampling:** Please provide the population size, respondent composition, inclusion criteria, response rate, and a stronger justification for the purposive sample of 150 participants.
4. **Measurement validity:** Cronbach's alpha establishes internal consistency but does not by itself demonstrate construct validity. Please provide additional information regarding questionnaire development/adaptation, content validation, and, where possible, construct validity assessment.
5. **Normality testing:** The Kolmogorov–Smirnov testing procedure and decision rule should be carefully rechecked. Please report the relevant statistics and p-values and reconsider the conclusion that all variables are normally distributed.
6. **Statistical consistency:** Please carefully verify Tables 3–9 against the original SPSS outputs. There appear to be inconsistencies, particularly in Table 4 and between Table 8 and its accompanying narrative regarding the artificial intelligence regression coefficient.
7. **Regression interpretation:** Regression coefficients such as β = 0.52 should not automatically be interpreted as a 52% increase in the dependent variable. Please revise these interpretations according to whether standardized or unstandardized coefficients are reported.
8. **Regression model:** Consider conducting a multiple regression including digital integration, artificial intelligence, and smart governance simultaneously. This would provide a stronger assessment of the unique contribution of each predictor and should be accompanied by appropriate diagnostic tests.
9. **Causal interpretation:** Given the cross-sectional, non-experimental design, causal terminology such as “directly affects,” “impact,” and “proves” should be moderated. The findings primarily demonstrate statistical associations or predictive relationships.
10. **Limitations:** Please add a dedicated limitations section addressing the single-company setting, purposive sampling, self-reported measures, cross-sectional design, potential common-method bias, and limited generalizability.

The manuscript has a potentially useful empirical contribution and addresses a relevant topic. Nevertheless, correction of the statistical inconsistencies and clearer methodological reporting are necessary to ensure that the conclusions accurately reflect the evidence.

**Recommendation: Approved with Reservations.**

**Best regards,**

**Reviewer**

Is the work clearly and accurately presented and does it cite the current literature?

Partly

If applicable, is the statistical analysis and its interpretation appropriate?

Partly

Are all the source data underlying the results available to ensure full reproducibility?

Yes

Is the study design appropriate and is the work technically sound?

Partly

Are the conclusions drawn adequately supported by the results?

Partly

Are sufficient details of methods and analysis provided to allow replication by others?

Partly

Reviewer Expertise:

Urban and Regional Planning; Sustainable Urban Development; Urban Governance

I confirm that I have read this submission and believe that I have an appropriate level of expertise to confirm that it is of an acceptable scientific standard, however I have significant reservations, as outlined above.

# Reviewer report: Reviewer response for version 1

**DOI:** 10.5256/f1000research.193434.r525116

**Published:** 2026-10-07

**Authors:** Khoren Mkhitaryan

**Affiliations:** 1 Armenian State University of Economics, Yerevan, Armenia

This article examines the relationship between smart government applications operationalized through digital integration, artificial intelligence, and smart governance and production process redesign at Al Ittihad Food Industries Company.

 Based on questionnaire data collected from 150 managers, engineers, and production employees, the authors use descriptive statistics, Pearson correlation, and regression analysis to examine the proposed relationships. The topic is relevant and timely, particularly given the increasing interaction between digital transformation, Industry 4.0, governance practices, and organizational process redesign. The manuscript also draws on recent literature and makes the underlying questionnaire data and survey instrument openly available, which is a positive contribution to transparency and reproducibility.

 Nevertheless, several methodological and interpretative issues need to be addressed before the conclusions can be considered fully supported by the evidence. In my view, the following points are particularly important for improving the scientific soundness of the article.

**1. Clarify the conceptual relationship between “smart government” and firm-level digital transformation.**

 The manuscript defines smart government primarily as a public-sector concept involving digital government, smart governance, AI, data-driven administration, transparency, and public services. However, the empirical study is conducted within a private industrial company, and several measured dimensions appear closely related to enterprise digital transformation and Industry 4.0 practices. The authors should therefore explain more explicitly how smart government practices or policies are transmitted to, and operationalized within, the internal production processes of the company. The theoretical model should distinguish government-level practices and policies from firm-level technological and organizational capabilities. If the questionnaire measures predominantly internal organizational practices, the terminology and theoretical framing should be reconsidered accordingly.

**2. Align the research design with the claims made in the manuscript.**

 The study uses a cross-sectional questionnaire and purposive sampling within a single company. Such a design can provide evidence of associations between the variables, but it does not by itself establish causality. The manuscript nevertheless repeatedly uses expressions such as “impact,” “direct effect,” “causal relationship,” and, in the Discussion, states that the researchers “experimentally examined” the relationship. The study is not experimental. Unless the authors can provide an appropriate causal identification strategy, these statements should be revised throughout the Abstract, Results, Discussion, and Conclusions. Expressions such as “is positively associated with,” “is related to,” or “statistically predicts” would more accurately reflect the research design.

**3. Clarify the stated mixed-methods design and the role of the interviews.**

 The manuscript states that a questionnaire and semi-structured interviews were used and subsequently describes the research as adopting a “mixed methodology.” However, the Methods section refers to questionnaire responses as “qualitative data,” while the reported analyses are quantitative. Moreover, sufficient information concerning the semi-structured interviews. the number and selection of participants, interview protocol, analytical/coding procedure, and resulting qualitative findings is not provided. The authors should either fully describe and report the qualitative component according to appropriate mixed-methods standards or remove the mixed-methods/interview claims and describe the study consistently as a quantitative observational survey.

**4. Strengthen the evidence for measurement validity.**

 Cronbach’s alpha values are reported and provide evidence concerning internal consistency. However, Cronbach’s alpha is a reliability measure and should not by itself be presented as evidence of construct validity. The authors should explain the origin and development/adaptation of the questionnaire items and provide appropriate evidence supporting content and construct validity. Where feasible, factor-analytic evidence should be provided to demonstrate that the items adequately represent the proposed dimensions of smart government practices and production process redesign.

**5. Reconsider and clarify the normality assessment and statistical assumptions.**

 The description and interpretation of the Kolmogorov–Smirnov procedure require careful revision. In particular, the decision rule reported in the manuscript should be verified and explained using the appropriate test statistic and significance criterion. More generally, the suitability of Pearson correlations and linear regression should not be justified solely by the marginal distribution of the study variables. The authors should also consider and report the relevant regression assumptions, including linearity, homoscedasticity, independence of residuals, and residual diagnostics where appropriate.

**6. Recheck the regression analyses and statistical reporting. This point must be addressed.**

 There appear to be inconsistencies in the reported regression statistics that require verification against the underlying data and SPSS output. In a simple linear regression with one predictor, the reported *t* and *F* statistics should have a mathematically consistent relationship, but several values in Tables 6–9 do not appear to be mutually consistent. There is also an apparent discrepancy in Table 8 between the reported coefficient and the coefficient discussed in the accompanying text. I strongly recommend that the authors re-run or carefully verify all regression analyses and report, for each model, the unstandardized coefficient (B), standard error, standardized coefficient (β), *t*, *F*, degrees of freedom, exact or appropriately formatted *p* values, R², and preferably confidence intervals.

 The interpretation of the regression coefficients should also be corrected. For example, β = 0.52 does not mean that a one-unit increase in the predictor produces a “52% increase” in the outcome. If β is standardized, it represents a change expressed in standard-deviation units; if an unstandardized coefficient is reported, its interpretation should be expressed in the original measurement units. Similar percentage interpretations elsewhere in the regression results should therefore be revised.

**7. Consider the analytical treatment of the three smart-government dimensions.**

 Digital integration, artificial intelligence, and smart governance are examined through separate correlations and simple regressions. These analyses demonstrate their individual associations with process redesign but do not establish their unique contributions when the other dimensions are controlled. If the authors wish to compare or discuss the independent contribution of these dimensions, a multiple regression model including the dimensions simultaneously would be more informative, accompanied by appropriate multicollinearity diagnostics.

**8. Discuss sampling, generalizability, and common-method limitations more explicitly.**

 The sample consists of 150 purposively selected respondents from one company. The authors should provide additional information about the sampling frame, eligibility criteria, population size and response rate, where available, and relevant respondent characteristics. Conclusions should be restricted to the population and organizational context actually studied. In addition, both predictor and outcome variables appear to have been measured from the same respondents using the same questionnaire at the same point in time. The possibility of common-method bias should therefore be acknowledged and, if possible, assessed.

**9. Improve consistency and precision in tables and statistical terminology.**

 All tables should be carefully checked against the statistical output and accompanying narrative. Statistical significance should normally be reported as *p* < .001 rather than *p* = .000. Table headings, coefficients, percentages, and textual interpretations should be checked for consistency. A careful language and technical edit would also improve clarity and readability.

**Concluding assessment**

 The manuscript addresses a relevant research topic and contains potentially useful empirical evidence concerning digital practices and production process redesign in an industrial setting. The availability of the underlying dataset and questionnaire is particularly welcome. However, the manuscript currently overstates what can be inferred from its cross-sectional design, and several aspects of the methodology and statistical reporting require clarification or correction. In particular, **the regression analyses and their interpretation, the causal/experimental language, the conceptual distinction between smart government and enterprise-level digital transformation, and the unclear mixed-methods component should be addressed before the article can be considered scientifically sound**. These issues appear potentially addressable through substantial revision, clarification, and reanalysis rather than requiring a fundamentally new study.

Is the work clearly and accurately presented and does it cite the current literature?

Partly

If applicable, is the statistical analysis and its interpretation appropriate?

Partly

Are all the source data underlying the results available to ensure full reproducibility?

Yes

Is the study design appropriate and is the work technically sound?

Partly

Are the conclusions drawn adequately supported by the results?

Partly

Are sufficient details of methods and analysis provided to allow replication by others?

Partly

Reviewer Expertise:

Management; Organizational Change and Transformation; Digital Transformation; Artificial Intelligence in Management; Business Process Management and Redesign; Smart Governance; Public Management; Socio-Economic Statistics; Research Methodology.

I confirm that I have read this submission and believe that I have an appropriate level of expertise to confirm that it is of an acceptable scientific standard, however I have significant reservations, as outlined above.

# Reviewer report: Reviewer response for version 1

**DOI:** 10.5256/f1000research.193434.r485689

**Published:** 2026-05-19

**Authors:** Rakibul Hasan Chowdhury

**Affiliations:** 1 Trine University, Angola, Indiana, USA

This manuscript investigates the impact of smart government practices, specifically digital integration, artificial intelligence, and smart governance, on production process redesign within an industrial company (Al Ittihad Food Industries Company). Using a descriptive-analytical methodology, the study collects data from 150 managers, engineers, and production employees through a structured questionnaire.

 The authors employ statistical methods including reliability analysis, normality testing, Pearson correlation, and regression analysis. The findings reveal significant positive relationships between smart government dimensions and production process redesign outcomes, including performance, quality, and organizational factors.

 The study concludes that smart government practices significantly contribute to enhancing industrial efficiency, improving production quality, and supporting organizational transformation aligned with Industry 4.0 principles.

Is the work clearly and accurately presented and does it cite the current literature?

Yes

If applicable, is the statistical analysis and its interpretation appropriate?

Yes

Are all the source data underlying the results available to ensure full reproducibility?

Yes

Is the study design appropriate and is the work technically sound?

Yes

Are the conclusions drawn adequately supported by the results?

Yes

Are sufficient details of methods and analysis provided to allow replication by others?

Yes

Reviewer Expertise:

NA

I confirm that I have read this submission and believe that I have an appropriate level of expertise to confirm that it is of an acceptable scientific standard.
